# Efficacy of 0.5M mannitol as an adjuvant to lidocaine and epinephrine for intra-oral nerve blocks - a split-mouth, randomized controlled trial

**DOI:** 10.1186/s12903-026-08468-3

**Published:** 2026-05-09

**Authors:** Radhika Singh, Anupam Singh, Mehul Saha, Adarsh Kudva, Srikanth Gadicherla, Kalyana C. Pentapati, A. Chitra, Dharnappa Poojary, Sreea Roy

**Affiliations:** 1 Department of Oral & Maxillofacial Surgery, Manipal College of Dental Sciences, Manipal Academy of Higher Education (MAHE), Manipal, India; 2 Department of Public Health Dentistry, Manipal College of Dental Sciences, Manipal Academy of Higher Education (MAHE), Manipal, India; 3 Department of Oral & Maxillofacial Surgery, Manipal College of Dental Sciences Mangalore, Manipal Academy of Higher Education (MAHE), Manipal, India

**Keywords:** Epinephrine, Dental procedure, Nerve block, Lidocaine, Local anesthesia, Mannitol

## Abstract

**Background:**

In dentistry operations, addition 0.5 M mannitol to lidocaine-epinephrine improves anesthesia efficacy. However, its effect on hemodynamic parameters and post-operative outcomes have not been evaluated. This study aimed to compare the efficacy, hemodynamic parameters, and post-operative outcomes of 2% lidocaine and 1:200,000 epinephrine, with or without 0.5 M mannitol, in intra-oral nerve blocks.

**Materials and methods:**

This prospective, randomized, controlled, triple-blind, split-mouth study included 25 patients who required intra-oral block for elective extraction of lower erupted teeth. The Test side (*n* = 25) received lidocaine-epinephrine-mannitol, while the Control side (*n* = 25) received lidocaine-epinephrine. The primary outcome measures were evaluated were onset and duration of anesthesia. The secondary outcome measures were pain (assessed with visual analogue scale [VAS] on day 0–3, and 7); hemodynamic parameters (including heart rate, systolic blood pressure [SBP], diastolic blood pressure [DBP], and oxygen saturation, assessed pre-, intra-, and post-operatively); and post-operative complications (swelling and trismus assessed on day 7).

**Results:**

The Test side had a significantly early onset of action (*p* = 0.006) and delayed return of sensation (*p* = 0.001). On day 0, VAS score was significantly lower in the Test side (*p* = 0.017), with no difference at other intervals (*p* > 0.05). Post-operatively, mouth opening was significantly greater on the test side (*p* = 0.023), with no difference in post-operative swelling (*p* = 0.317). The control side had a significantly higher intra- (*p* = 0.018) and post-operative (*p* = 0.006) heart rate, with comparable SBP, DBP, and oxygen saturation.

**Conclusion:**

Lidocaine-epinephrine-mannitol formulation showed improved anesthetic efficacy, reduced pain on the day of procedure, stable hemodynamic parameters, and increased post-operative mouth opening.

**Trial registration:**

CTRI/2022/10/046921 [Registered on: 31/10/2022].

**Supplementary Information:**

The online version contains supplementary material available at 10.1186/s12903-026-08468-3.

## Introduction

Intra-oral nerve blocks are common techniques for achieving anesthesia for minor oral surgical procedures or extractions. Among the various local anesthetics, 2% lidocaine with epinephrine has been widely used in dental procedures due to its rapid onset, effective pain management, and prolonged duration of action, attributed to vasoconstrictive effects of epinephrine [1]. However, their success is inconsistent, with failure rates ranging from 10 to 39% [2].

One of the potential causes of failure is the perineural barrier, which limits the diffusion of anesthetic solutions into the nerve trunk. The tight junctions along the inner layer of perineurium can prevent the diffusion of both hydrophilic and lipophilic compounds [3–6]. These diffusion barriers render a large fraction of the injected local anesthetic ineffective and can potentially lead to anaesthetic failure.

To further improve the performance of lidocaine with epinephrine, various adjuvants have been explored. The use of hyperosmolar solutions like 0.5 M mannitol as adjuvants have been reported. Mannitol facilitates better penetration of the anesthetic agent in nerves [7]. A 0.5 M mannitol solution has been found to be effective in facilitating perineurial membrane opening, thereby enhancing their permeability to macromolecules and/or ions [8]. Based on this mechanism, 0.5 M mannitol is reported to increase the efficacy of local anesthetic agents in intra-oral nerve blocks [8–11]. Addition of mannitol to local anesthetics has been demonstrated to prolong the duration of anesthesia, and improve the pain control during tooth extraction, thereby improving patient experience [9, 12].

Hemodynamic parameters like blood pressure and heart rate are key indicators of cardiovascular response to local anesthetics, particularly with epinephrine [13]. Mannitol as an adjuvant may influence these parameters, but its specific effects with lidocaine and epinephrine remain unclear [11]. Moreover, evaluating post-operative parameters including healing, and discomfort at injection site is crucial in evaluating anesthetic efficacy.

The literature review indicated that the randomized controlled trials (RCTs) assessing the efficacy of lidocaine with epinephrine, with or without 0.5 M mannitol, are limited to inferior alveolar nerve (IAN) blocks and does not necessarily address hemodynamic parameters or postoperative outcomes [9, 10, 14, 15]. Considering the existing evidence, the present study was designed to evaluate and compare the efficacy of lidocaine with epinephrine administered with and without 0.5 M mannitol as an adjuvant in intra-oral nerve blocks. The primary objective was to assess and compare the onset and duration of anesthesia achieved with the addition of 0.5 M mannitol versus the conventional lidocaine with epinephrine alone. Secondary objectives included comparative evaluation of intra-operative pain, associated hemodynamic responses, and post-operative outcomes between the two anesthetic regimens. We hypothesized that addition of adjuvant 0.5 M mannitol would not produce any significant difference in any of the above parameters.

## Materials and methods

### Study design and ethics

This prospective, randomized, controlled, triple-blind, split-mouth single center study was performed over a period of 18 months (November 2022 to April 2024) in the department of Oral and Maxillofacial Surgery at a tertiary care hospital. The study was approved by the Institutional Ethical Committee of the Institute (KH-IEC1: 81/2022, Dated: March 9, 2022) and registered with Clinical Trial Registry of India (CTRI/2022/10/046921). Prior to enrolment, written informed consent was obtained from all the patients. The design, conduct, analysis, and reporting of this randomized controlled trial adhered to the Consolidated Standards of Reporting Trials (CONSORT) guidelines.

### Patients population

The study included male and female patients, aged between 18 and 35 years, who required similar intra-oral block of IAN, or mental nerve, or infraorbital nerve (ION) bilaterally within the same arch, and were not allergic to lidocaine and/or mannitol. Only patients requiring extraction of non-symptomatic erupted teeth were included. The indications for extraction included grossly decayed teeth, teeth with failed root canal treatment, periodontally compromised teeth, or orthodontic extractions. Since this was a split mouth study, both the test and the control sides within the same patient had comparable tooth conditions, thereby minimizing the risk of bias. The patients with any underlying systemic disease, American Society of Anasthesiologists (ASA) Physical status Class 3 and above, patients on neurogenic pain medications, and pregnant or lactating mothers were excluded from the study.

All the patients underwent elective extraction of upper or lower erupted teeth, and the type of anesthesia was any of the intra-oral nerve block mentioned earlier. Intra-oral nerve block and forceps extraction were performed as per the standard guidelines by a single dental surgeon with at least 10 years of experience in performing the procedure. All the patients were administered injections by a single experienced oral and maxillofacial surgeon to minimize operator variability. The inferior alveolar nerve block (IANB) was administered using the conventional Halsted technique, with the pterygomandibular raphe and coronoid notch serving as standard anatomical landmarks. Similarly mental and infraorbital nerve blocks were performed using the standardized intra-oral landmarks. Injections were administered manually at a slow, consistent rate of approximately 1mL per minute. The consistent technique employed by a single operator across all participants on both sides of the split-mouth design served to minimize procedural variability.

### Study groups

The groups included: the Test side (received a mixture of 0.5 M Mannitol, 2% lidocaine, and 1:200,000 epinephrine), and the Control side (received 2% lidocaine and 1:200,000 epinephrine).

### Study procedure

In this study, a 0.5 M mannitol solution was prepared using the following calculations: The molecular weight of mannitol is 182.172. Commercially available 20% mannitol (Otsuka Pharmaceuticals India Private Limited) contains approximately 1.098 M (200/182.172 ≈ 1 mol per bottle). To achieve a 0.5 M concentration, an equal dilution with normal saline was performed (e.g., 1 ml of 20% mannitol solution + 1 ml of normal saline = 0.5 M mannitol). All solutions were prepared immediately prior to administration (within 10 min time frame of LA administration) by a single trained dental surgical nurse, ensuring consistency across all patients and eliminating any potential variability which could have arisen due to preparation timing.

The Test side received 1.5 ml of 2% lidocaine and 1:2,00,000 epinephrine (Lox 2%, Manufactured by Neon Laboratories) mixed with 0.9 ml of 0.5 M mannitol. For the Control side, a mixture of 1.5 ml of 2% lidocaine and 1:2,00,000 epinephrine mixed with 0.9 ml of normal saline (as a placebo) was used. The solutions were withdrawn from standard dental syringes into a 3 ml Luer-Lok disposable syringe (Unilok, Hindustan Syringes & Medical devices Ltd) fitted with a 24G × 25 mm needle. The anesthetic solutions were prepared by a single dental surgical assistant (DSA) and handed over to the dental surgeon, labeled with computer-generated randomization codes. Each patient received one cartridge per group. Following administration of the anesthetic solution via the scheduled intra-oral nerve block, the injection time and the onset of tingling sensation in the region anaesthetized were recorded.

### Randomization and blinding

Patients were randomized into the test and control groups using a computer-generated randomization sequence by a resident. The randomized sequences, indicating the group allocation, were handed over in a sealed envelope to the DSA involved in handling the local anaesthetic preparation. The dental surgeon, the patient and the outcome assessor were blinded to the intervention. Another trained examiner recorded the outcome measures and tabulated them in different anonymized groups as per the randomization codes in Microsoft Excel sheet (Microsoft 365, Microsoft Corp., Redmond, WA, USA).

### Outcome measures

#### Primary outcome

For each side, the onset of action LA was determined by asking the patient about the first symptom of tingling sensation after administration of the injection. The duration of anesthesia was determined by the return of sensation on probing.

#### Secondary outcomes

Intra-operative pain was recorded, if it was reported by the patient by using an 11-point visual analogue scale (VAS). Post-operative pain was assessed on Days 0, 1, 2, 3, and 7 using the same VAS. The scale ranged from 0 to 10, with 0 and 10 suggesting no pain and worst imaginable pain, respectively. These scores were self-recorded by the patient in a scoring sheet administered to the patients on the day of procedure and were handed over to the dental surgeon on the day of follow-up visit.

Hemodynamic parameters, including heart rate (HR), systolic blood pressure (SBP), diastolic blood pressure (DBP), and oxygen saturation (SpO_2_), were assessed pre-operatively, intra-operatively (at the time of and after administering the local anesthetic), and 30-minutes post-operatively, using a digital pulse oximeter (Accusure Finger Pulse Oximeter, Microgene Diagnostic Systems Ltd) and digital sphygmomanometer (OMRON healthcare manufacturing Vietnam Co, Ltd).

Post-operative complications: On post-operative day 7, during the routine follow-up visit, the patients were evaluated for healing, the presence or absence of swelling, and trismus. Trismus was assessed by measuring pre- and post-operative mouth opening (inter-incisal distance between upper and lower central incisors) in millimeters using a scale, particularly for inferior alveolar nerve block.

The patients were scheduled for the procedure on the opposite side after one week, and the same protocol was followed. The one-week interval between two different procedures ensured elimination of any carry-over effect, since lignocaine has a short half-life (1.5 to 2 h) and the osmotic effect of mannitol on perineural membranes are transient. If anesthesia was not achieved after the initial administration of the local anesthetic, the combination was not re-administered. After the surgery, the patient was prescribed the same antibiotics and analgesics (Tab. Amoxicillin-Clavulanate 625 mg b.d. and Tab. Paracetamol 650 mg t.i.d. for 3 days).

### Sample size calculation

Based on the findings of Pathak et al., [9] an effect size of 1.1 was obtained for duration of anesthesia. Substituting this value, with 90% power and 95% confidence interval, the sample size obtained was 19. Considering 20% attrition, the sample size was inflated and rounded to 25.

### Statistical analyses

The data was analyzed with SPSS (IBM, Armonk, NY, USA) version 23.0 for Windows. Normality was tested using Shapiro – Wilks test which showed that data was non-normal in distribution. Comparison of categorical variables was done using Wilcoxon signed rank test owing to the split mouth study design. Effect size values (Rank biserial correlation) were reported for all the outcomes. Owing to the multiple comparisons across different time points within each outcome, Holm–Bonferroni correction was applied. Effect size values (Rank biserial correlation) were reported for all the outcomes.

## Results

**Fig. 1:**
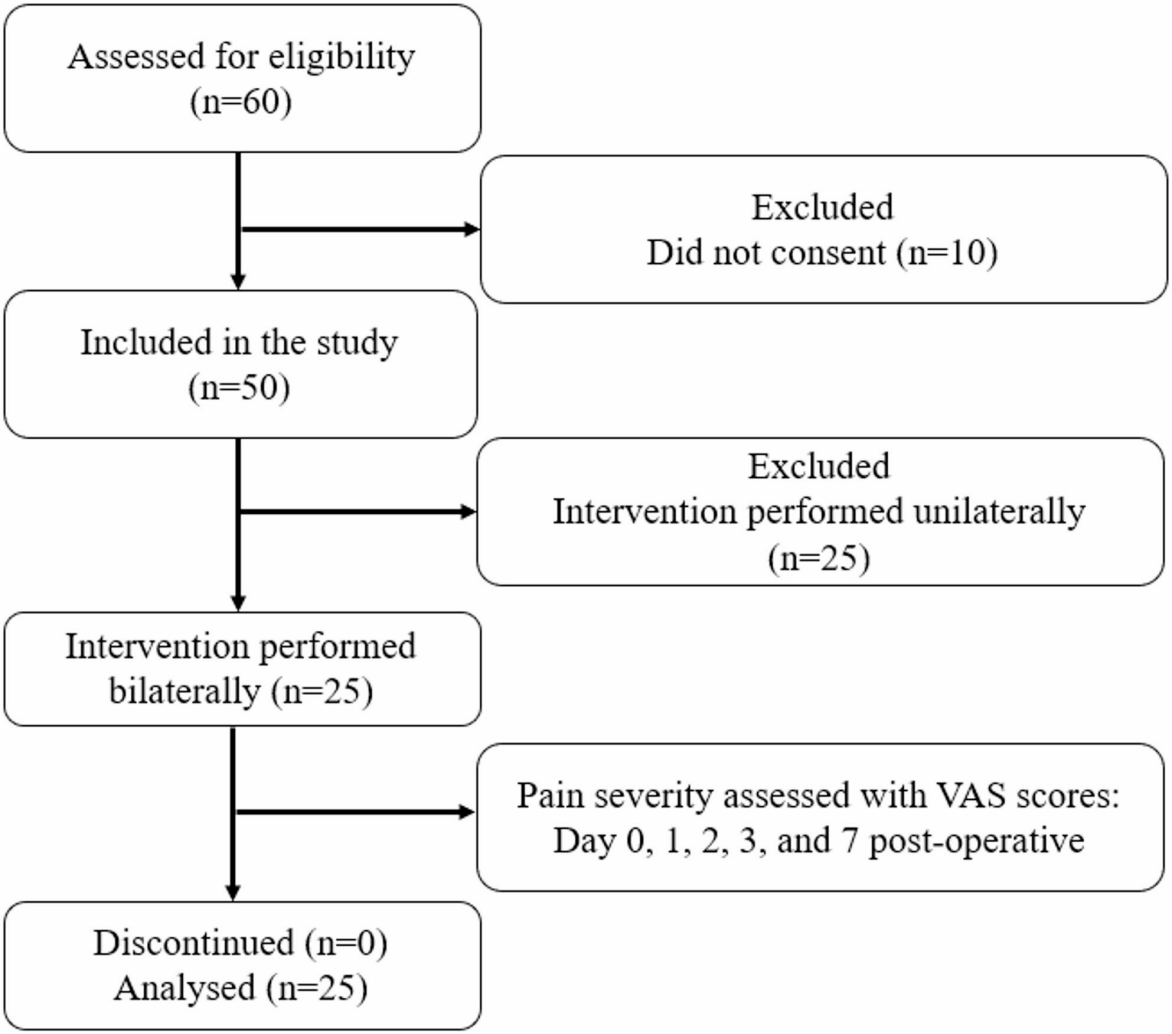
Study flowchart depicting the patient recruitment and exclusion

A total of 68 patients were screened and 60 fulfilled eligibility criteria. Of these 60 patients, 10 did not sign informed consent form and thus were excluded. Of 50 patients included in the study, bilateral intervention was performed in only 25, while remaining 25 received only unilateral intervention, as they did not return for second intervention and thus were excluded. Finally, all 25 patients who received bilateral intervention, completed the study and were included in the analysis (Fig. 1).

The mean age of participants was 22.28 ± 5.54 years with almost similar distribution of males (*n* = 13) and females (*n* = 12) (Table 1). The Test side had a significantly early onset of action (*p* = 0.006) with a significantly delayed return of sensation (*p* = 0.001) when compared to the control side (Table 2). None of the patients in either side reported intra-operative pain.

**Table 1:** Demographic distribution of the enrolled participants

Variables	Value
Total Number of participants	25
Mean Age (years)	22.28 ± 5.54
Median (years)	21
Gender Distribution	
Males	13 (52%)
Females	12 (48%)

**Table 2:** Comparison of onset and return of sensation after administration of local anesthesia

Variables	Test side (*n* = 25)	Control side (*n* = 25)	*p*	Effect size (*r*)
Onset LA action (Sec)	34.44 ± 16.57	43.80 ± 20.53	0.006	0.65
Return of sensation (min)	126.00 ± 30.00	99.60 ± 29.65	0.001	0.84

*LA* Local anesthesia, Effect size Interpretation – 0.1 small; 0.3 medium, 0.5 large

On day 0, the mean VAS score was significantly lower on the Test side compared to the Control side (*p* = 0.017). However, the Test and the Control sides had comparable mean VAS score at other intervals (*p* > 0.05) (Table 3).

**Table 3:** Comparison of post-operative pain. For post-operative pain assessment at five time points, multiple comparisons between test and control sites were performed with Wilcoxon signed-rank test followed by Holm–Bonferroni correction. None of the time points showed a statistically significant difference between test and control

VAS score	Test side (*n* = 25)	Control side (*n* = 25)	*p*	Effect size (*r*)
Day 0	2.76 ± 2.96	3.56 ± 3.15	0.017	0.59
Day 1	1.88 ± 2.52	2.24 ± 2.57	0.274	0.32
Day 2	1.32 ± 2.10	1.28 ± 1.97	0.785	0.13
Day 3	0.92 ± 1.68	0.76 ± 1.30	0.674	0.19
Day 7	0.00 ± 0.00	0.20 ± 0.50	-	-

There was no significant difference in the mean SBP, DBP, and SpO_2_ at any of the intervals between the Test and Control sides. There was significantly higher HR in the Control side during the procedure (*p* = 0.018) and in the post-operative period (*p* = 0.006) (Table 4).

**Table 4:** Comparison of hemodynamic assessment. For SBP, DBP, and SPO_2_ at three time points, multiple comparisons between test and control sites were performed with Wilcoxon signed-rank test followed by Holm–Bonferroni correction. None of the time points showed a statistically significant difference between test and control even before and after applying Holm–Bonferroni correction. However, heart rate assessed at three time points, intra-operative and post-operative assessment showed statistically significant differences between test and control sites after applying Holm–Bonferroni correction

	Variables	Test side (*n* = 25)	Control side (*n* = 25)	p	Effect size (r)
Pre-operative	SBP	118.56 ± 7.77	120.72 ± 7.19	0.073	0.51
	DBP	75.92 ± 5.73	76.32 ± 5.65	0.792	0.1
	HR	78.84 ± 7.45	78.08 ± 6.40	0.986	0
	SPO_2_	98.76 ± 0.72	98.84 ± 0.80	0.527	0.2
Intra-operative	SBP	124.40 ± 8.21	126.00 ± 7.22	0.354	0.27
	DBP	78.00 ± 4.08	77.60 ± 4.36	0.763	0.09
	HR	80.44 ± 6.99	84.76 ± 8.17	0.018	0.56
	SPO_2_	98.76 ± 0.66	98.76 ± 0.78	0.999	0
Post-operative	SBP	117.64 ± 9.62	121.00 ± 8.78	0.132	0.39
	DBP	75.60 ± 6.51	76.00 ± 6.45	0.805	0.07
	HR	77.28 ± 8.11	80.32 ± 5.83	0.006	0.649
	SPO_2_	99.00 ± 0.76	98.92 ± 0.86	0.617	0.14

*DBP* Diastolic blood pressure, *HR* Heart rate, *SBP* Systolic blood pressure, *SPO*_*2*_ Oxygen Saturation

Though none of the patients had trismus during post-operative intervals, the mouth opening was significantly higher in the Test side than the Control side (*p* = 0.023). No significant differences were seen with respect to post-operative swelling (*p* = 0.317) (Table 5). No complications were encountered during the study period in any of the patients.

**Table 5:** Comparison of pre- and post-operative mouth opening and post-operative swelling

	Variables	Test side (*n* = 25)	Control side (*n* = 25)	*p*	Effect size (*r*)
Mouth opening	Pre-operative	42.92 ± 3.67	42.64 ± 3.73	0.102	1
	Post-operative	42.56 ± 3.81	42.00 ± 3.38	0.023	1
Post-operative swelling		0.20 ± 0.41	0.28 ± 0.46	0.317	0.5

To account for multiple comparisons across time points within each outcome, Holm–Bonferroni correction was applied. Following adjustment, no statistically significant differences were observed for pain, systolic blood pressure, diastolic blood pressure, or oxygen saturation at any time point. However, intra-operative and post-operative heart rate showed statistically significant differences after correction. Also, post-operative mouth opening remained significant.

## Discussion

The principal findings of our study suggested that the addition of 0.5 M mannitol to 2% lidocaine and 1:200,000 epinephrine led to a significantly early onset of action and delayed return of sensation, signifying prolonged duration of action. Pathak et al. reported that use of 0.5 M mannitol as an adjuvant to 2% lidocaine led to a significant improvement in the onset and duration of anesthesia compared to lidocaine alone or with 1:80000 epinephrine [9] Wolf et al., [10] and Smith et al. [14] demonstrated that the pulpal anesthesia was significantly higher with a combination of lidocaine + epinephrine + mannitol compared to the combination of lidocaine-epinephrine. Kreimer et al. demonstrated that a combination of lidocaine-epinephrine-mannitol led to a 26% higher success rate compared to combination of lidocaine and epinephrine [11].

The enhanced efficacy can be attributed to various actions, including osmotic properties of mannitol that increases the nerve membrane permeability, thus permitting a quicker uptake of lidocaine into the nerve fibers [10]. Moreover, the vasoconstrictive action of epinephrine results in prolonged anesthetic action by decreasing the systemic absorption and maintaining higher concentrations at the site of action [16].

Mannitol is a naturally occurring sugar alcohol that is used primarily for its osmotic diuretic properties. It also acts as a scavenger of oxygen-derived free radicals, that can limit cellular edema and reduce renal tubular injury. It is the preferred drug for management of raised intracranial pressure (ICP), for renal protection during cardiac, vascular, and renal transplantation surgeries. As an osmotic diuretic, mannitol promotes the movement of water from intracellular and interstitial compartments into the intravascular space [7, 17].

Local anesthetic agents act primarily by inhibiting influx of sodium ions through voltage-gated sodium channels present in the neuronal cell membrane, thereby preventing action potential generation and propagation. Despite their widespread use, intra-oral nerve blocks are associated with significantly high failure rates [18]. One widely proposed mechanism for high failure rate is the perineural barrier, which may limit diffusion of the anesthetic solution into the nerve trunk [19, 20]. Various techniques and adjuvants have been used to increase the success rate and efficacy the intra-oral blocks [21, 22]. Antonijevic et al. demonstrated that 0.5 M mannitol solution effectively increases perineural membrane permeability, facilitating enhanced permeability and penetration of macromolecules and ions [8]. By increasing the perineural permeability and potentially influencing nerve conduction, mannitol can improve the success rate and efficacy of intra-oral nerve blocks when administered in combination with the local anesthetic agents [8].

Substantial variations have been reported with respect to the pain in literature. Regarding solution deposition pain, few studies have shown that mannitol formulations lead to a significant reduction in pain [23], while others have demonstrated that no difference between formulations with and without mannitol [10, 14]. In the present study, none of the patients reported intra-operative pain during the procedure on either side. Regarding post-operative pain, the mannitol resulted in significantly lower self-recorded VAS scores on Day 0, with no subsequent difference noted in subsequent days (day 1, 2, 3, and 7). It is important to note that this Day 0 score reflects post-operative pain and should not be interpreted as a measure of intra-operative pain or discomfort. Compared to a lidocaine-epinephrine formulation, lidocaine-epinephrine-mannitol formulation is reported to significantly reduce the pain score during the procedure [11], and on the day of procedure [12]. On the contrary, another study demonstrated that significantly greater proportion of patients receiving formulation without mannitol were pain free at day 0 and day 3, with comparable pain relief at Day 1 and 2 [16]. However, various authors reported that formulations with and without mannitol were comparable regarding post-procedure pain from day 0 to day 3 [10, 14, 24].

In our study, the addition of 0.5 M mannitol did not have significant effect on hemodynamic parameters, except significant reduction in intra- and post-operative HR, though the reduction was within normal range. Use of lidocaine with epinephrine, a frequently used anesthetic agent in dentistry, results in increased HR and myocardial contractility by acting on β-adrenergic receptors [24]. The effects of epinephrine on the cardiovascular system are well-known, with various authors demonstrating a rise in HR and cardiac output after the administration of local anesthetic agent [24, 25]. However, the effect of lidocaine and epinephrine on SBP and DBP is conflicting with some suggesting no significant change [26], while other demonstrating significant rise in SBP and DBP following administration of lidocaine-epinephrine [27]. These hemodynamic effects are transient [24], and vital parameters return to baseline following the procedure.

While the rise in HR and BP may be attributed to fear of injections or dental procedure, addition of mannitol may attenuate this effect on HR through a mechanism related to its osmotic activity, which can change the pharmacokinetics of lidocaine-epinephrine. As described above, mannitol increases the diffusion of local anesthetics through the tissue, leading to a more successful block with less epinephrine absorbed systemically [11, 27]. As less epinephrine would enter the systemic circulation, the HR would drop while still providing sufficient anesthesia for the procedure, potentially leading to a decreased cardiovascular response. This justifies our findings of significantly lower HR with lidocaine-epinephrine-mannitol compared to lidocaine-epinephrine, with no significant difference with regards to SBP, DBP, and SpO_2_. However, it is important to note that while this difference was statistically significant, the absolute HR values on both the test and control sides remained within normal physiological limits throughout the study period. Thus, clinical significance of this difference is limited, and the addition of mannitol to the anesthetic formulation did not adversely affect the hemodynamic stability.

Literature suggests that inferior alveolar nerve block with 2% lidocaine and 1:100,000 epinephrine is associated with 3–9% incidence of post-operative trismus [28, 29]. Wolf et al. [10] reported that 2–5% patients had trismus with lidocaine (36 mg) + epinephrine (18 µg), lidocaine (36 mg) + epinephrine (18 µg) + mannitol (0.5 M), and lidocaine (63.6 mg) + epinephrine (32 µg) + mannitol (0.5 M) [6]. Smith et al. observed a 12% and 10% incidence of trismus, at the time subjective numbness wore off, with the use of lidocaine (127.2 mg) + epinephrine (50 µg) and lidocaine (127.2 mg) + epinephrine (50 µg) + mannitol (0.5 M), respectively; with same incidence noted at day 1, decreased to 2% and 8% on day 2, and then increased to 17% and decreased to 5% on day 3, respectively [14]. Thus, they attributed high incidence of trismus to the increased amount of lidocaine (127.2 mg). Cohen et al. showed that patients receiving formulation of lidocaine (68.8 mg) + epinephrine (50 µg) + mannitol (0.9 M) had a higher incidence of trismus than those receiving formulation of lidocaine (68.8 mg) + epinephrine (50 µg) (10% vs. 0%) [11]. They ascribed the incidence of trismus to the use of high molar mannitol (0.9 M). In the present study, none of the patients developed trismus and the patients receiving formulation with mannitol had significantly larger post-operative mouth opening; however, this finding cannot be attributed to the use of mannitol, as 0.5 M solution is inert [10].

While several outcomes in this study achieved statistical significance, it is equally important to evaluate their clinical relevance. The approximately 26-minute prolongation in duration of anesthesia (*r* = 0.84) represents the most clinically meaningful finding, as extended anesthetic coverage reduces the likelihood of intra-operative pain and the need for supplemental injections, directly benefiting patient experience and operative efficiency. The earlier onset of anesthesia (*r* = 0.65), though modest in absolute seconds, may be particularly relevant in anxious or needle-phobic patients. Heart rate differences, while statistically significant and associated with moderate-to-large effect sizes, remained within normal physiological limits in both groups; however, the comparatively stable hemodynamic profile of the mannitol-containing formulation may hold clinical relevance for patients with underlying cardiovascular conditions. Post-operative mouth opening, though statistically significant, showed an absolute difference below the clinically recognized threshold for trismus, and both groups maintained functional mouth opening. Post-operative pain scores were comparable between groups after correction for multiple comparisons, and the Day 0 difference, even before correction, fell below the minimum clinically important difference (MCID) of 1.5–2.0 cm for VAS. These observations underscore the importance of interpreting statistical findings within their clinical context and highlight that the primary benefit of mannitol addition lies in anesthetic efficacy rather than post-operative pain management.

This study has several strengths. The randomized, split-mouth study design minimized inter-individual variability and allowed each patient to serve as their own control, thereby enhancing internal validity. Inclusion of multiple outcome measures related to the procedure and the hemodynamic parameters, provides a comprehensive assessment of the clinical performance and safety profile of lidocaine-epinephrine-mannitol formulation. In addition, extended follow-up for pain assessment and complications, and monitoring of cardiovascular parameters addresses gaps in the existing literature.

Despite the strengths, the study has certain limitations. The study was conducted at a single center with a relatively small sample size, which may limit generalizability of the study. Although all included patients completed bilateral interventions, a significant proportion of initially enrolled patients did not return for the second procedure, introducing a potential attrition bias. Although none of these patients reported any adverse effects related to the study interventions, their non-return was attributed to personal and logistical reasons. This attrition may have reduced the effective study power and limits the external validity of the study findings. With regards to standardization of injections, they were delivered manually rather than via a computer-controlled local anesthetic delivery (CLAD) device; therefore, precise standardization of injection speed and volume deposition rate could not be objectively quantified. Future studies incorporating CLAD devices may allow for more precise control of these variables and improve reproducibility.

Another limitation of the study was that the secondary outcomes were exploratory in nature, and no formal adjustment for multiple comparisons was performed. This may increase the probability of Type I error and should be considered while interpreting the findings. The study population was restricted to ASA I–II patients aged 18–35 years undergoing extraction of non-symptomatic erupted teeth. While this was a deliberate methodological choice to minimize confounding variables and establish pharmacological efficacy under controlled conditions, it limits direct extrapolation of findings to older patients, those with systemic comorbidities, or patients presenting with symptomatic or acutely inflamed teeth, populations in whom anesthetic failure is of greatest clinical concern. Future studies are warranted to evaluate the efficacy and safety of the mannitol-lidocaine-epinephrine formulation in these higher-risk and clinically challenging subgroups. Although no major adverse events were noted during the study period, the findings should be interpreted with some caution.

## Conclusion

Within the limits of the study, the addition of 0.5 M mannitol to a lidocaine-epinephrine formulation resulted in improved anesthetic efficacy in minor oral surgery, reduced pain on the day of procedure, and stable hemodynamic parameters with no adverse effects.

## Electronic Supplementary Material

Supplementary Material 1.

## Data Availability

The data set generated and analyzed during the study will be made available from the corresponding author on request from the publisher.

## Abbreviations

RCTs: Randomized controlled trials
IAN: Inferior alveolar nerve
CONSORT: Consolidated Standards of Reporting Trials
ION: Infraorbital nerve
ASA: American Society of Anasthesiologists
DSA: Dental surgical assistant
VAS: Visual analogue scale
HR: Heart rate
SBP: Systolic blood pressure
DBP: Diastolic blood pressure
SpO_2_: Oxygen saturation
ICP: Intracranial pressure
