# Supplementary material for: Efficacy of 0.5M mannitol as an adjuvant to lidocaine and epinephrine for intra-oral nerve blocks - a split-mouth, randomized controlled trial
Source: BMC Oral Health. 2026 May 9;26:1213. doi: 10.1186/s12903-026-08468-3 (PMC13343650; doi:10.1186/s12903-026-08468-3)
Supplement: Supplementary file 1 — Supplementary Material 1. [file 12903_2026_8468_MOESM1_ESM.docx]

| **Variables** | **Value** |
| --- | --- |
| Total Number of participants | 25 |
| Mean Age (years) | 24.34 ± 4.41 |
| Median (years) | 24 |
| Gender Distribution | |
| Males | 15 (60%) |
| Females | 10 (40%) |

Supplementary table 1: Demographic distribution of the Dropout participants.

Supplementary table 2: Comparison of demographic distribution of enrolled and dropout patients.

| V**ariables** | \| **Enrolled (n=25)** \|  \| \| --- \| --- \| | **Dropouts (n=25)** |
| --- | --- | --- | --- | --- |
| \| Mean Age (years) \| \| --- \| | 22.28 ± 5.54 | 24.34 ± 4.41 |
| \| Median Age (years) \| \| --- \| | 21 | 24 |
| \| Males \| \| --- \| \|  \| | 13 (52%) | 15 (60%) |
| Females | 12 (48%) | 10 (40%) |
